# A Hybrid Digital-4E Strategy for comorbid migraine and depression: a medical hypothesis on an AI-driven, neuroadaptive, and exposome-aware approach

**DOI:** 10.3389/fneur.2025.1587296

**Published:** 2025-05-29

**Authors:** Parisa Gazerani

**Affiliations:** Department of Life Science and Health, Faculty of Health Sciences, Oslo Metropolitan University, Oslo, Norway

**Keywords:** migraine-depression comorbidity, 4E cognition, digital health, AI-driven therapy, closed-loop systems, exposome, precision medicine, neuroadaptive

## Abstract

**Objective:**

The co-occurrence of migraines and depression presents a critical clinical challenge, affecting up to 50% of individuals with either condition. This comorbidity leads to greater disability, higher healthcare costs, and poorer treatment outcomes than either disorder alone. Despite a bidirectional pathophysiological relationship, current models remain static and fragmented, treating each condition separately. This paper proposes a Hybrid Digital-4E Strategy, deployed on an AI-driven neuroadaptive digital health platform, integrating closed-loop therapy, digital phenotyping, and exposome tracking to enable real-time, personalized care.

**Methods:**

Grounded in the 4E cognition framework (Embodied, Embedded, Enactive, and Extended cognition), this strategy reconceptualizes migraine-depression as an interactive system rather than two separate conditions. The platform integrates real-time biomarker tracking, neuromorphic AI, and precision environmental analytics to dynamically optimize treatment. Adaptive chronotherapy, brain-computer interfaces (BCIs), and virtual reality (VR)-based neuroplasticity training further enhance intervention precision.

**Conclusion:**

A closed-loop, AI-driven neuroadaptive system could improve outcomes by enabling early detection, real-time intervention, and precision care tailored to individual neurophysiological and environmental profiles. Addressing AI bias, data privacy, and clinical validation is crucial for implementation. If validated, this Hybrid Digital-4E Strategy could redefine migraine-depression management, paving the way for precision neuropsychiatry.

## 1 Introduction

Migraine and depression are among the most prevalent and debilitating neurological and psychiatric disorders worldwide, collectively affecting hundreds of millions of individuals (1). Epidemiological studies indicate a strong bidirectional relationship (2), with 30–50% of individuals diagnosed with either condition also experiencing the other (3). This high rate of comorbidity suggests overlapping pathophysiological mechanisms, including genetic predisposition, neurotransmitter dysregulation (serotonin, dopamine, CGRP), neuroinflammation, autonomic dysfunction, and abnormalities in the hypothalamic–pituitary–adrenal (HPA) axis (4–6). Despite this shared neurobiology, current clinical frameworks predominantly treat migraine and depression as distinct disorders, leading to suboptimal patient outcomes, prolonged disease burden, and a continued reliance on trial-and-error pharmacological strategies (6, 7).

A comorbid approach to treatment offers an opportunity to rethink existing management paradigms (8–10). However, this dual-focus strategy also presents challenges: therapies designed to alleviate one condition may inadvertently exacerbate the other. For instance, selective serotonin reuptake inhibitors (SSRIs) and serotonin-norepinephrine reuptake inhibitors (SNRIs), while effective for depression, may lower the threshold for migraine attacks (11), whereas certain migraine-specific treatments such as CGRP antagonists can negatively impact mood regulation (12). This highlights the need for a precise, dynamic, and personalized treatment approach, one that accounts for the intricate interplay between migraine and depression (4) rather than treating them in isolation (4, 7).

### 1.1 Limitations of existing approaches

Current treatment strategies primarily rely on pharmacotherapy (13), including SSRIs, SNRIs, tricyclic antidepressants (TCAs) (14), and CGRP blocking agents (12, 15). However, these treatments often suffer from limited efficacy, significant side effects, and poor adherence (16, 17) due to the highly individualized nature of both disorders. Non-pharmacological interventions, such as cognitive-behavioral therapy (CBT), biofeedback, lifestyle modifications, and mindfulness-based techniques have demonstrated promise (18) but are often reactive rather than adaptive, lacking the ability to adjust dynamically to symptom fluctuations and neurophysiological changes (19).

A fundamental limitation of existing approaches is their static, retrospective, and fragmented nature. Traditional models fail to account for the continuous interplay of biological, behavioral, and environmental factors that influence the course of migraine-depression comorbidity (4, 8). The biopsychosocial model, while conceptually valuable (20, 21), remains largely descriptive rather than actionable in real-time interventions (22). Additionally, digital health solutions, such as Migraine Buddy, Mindstrong, and mood-tracking applications (23, 24), are primarily passive tools, offering users retrospective symptom analysis rather than AI-driven, adaptive interventions. Wearable technologies (e.g., Fitbit, WHOOP, Apple Health) collect physiological and behavioral data (25), yet they lack the neuroadaptive capabilities required for precision treatment adjustments (26–28). To address these limitations, this paper proposes a Hybrid Digital-4E Strategy, integrating AI-driven (29, 30) closed-loop therapy, digital phenotyping (31), and exposome tracking to enable a real-time, adaptive, and personalized treatment approach (32).

### 1.2 4E cognition as a theoretical framework for migraine-depression therapy

This framework is grounded in 4E cognition (33), which posits that cognition is Embodied, Embedded, Enactive, and Extended. This paradigm shift is particularly relevant for migraine-depression comorbidity (6, 7), as it accounts for the dynamic interactions between neurophysiological processes, behavioral patterns, and environmental influences. Embodied cognition recognizes that neurophysiological states (pain perception, mood regulation) are deeply tied to bodily processes (34). Embedded cognition (35) emphasizes the role of external factors (e.g., circadian disruption, environmental stressors, diet, pollution) in shaping symptoms and treatment responses. Enactive cognition (36) suggests that symptoms and behaviors are not merely reactive but actively shape disease progression, necessitating an adaptive therapeutic model. Extended cognition (37) demonstrates how external tools (e.g., digital health platforms, AI-driven interventions, brain-computer interfaces) can augment treatment strategies beyond traditional clinical models.

By integrating real-time biomarker tracking, neuroadaptive treatment strategies, and precision environmental analytics, this model moves beyond static monitoring to offer dynamic, AI-driven interventions that can personalize both pharmacological and non-pharmacological treatments (38, 39).

### 1.3 The role of the exposome in adaptive therapy

A key innovation of this approach is the incorporation of the exposome (40, 41), the cumulative effect of an individual’s lifetime environmental exposures (e.g., air pollution, urban stressors, diet, circadian misalignment, temperature fluctuations) on disease progression and treatment response. Unlike conventional models that rely solely on internal biomarkers, exposome integration allows for environmentally responsive treatment strategies, bridging the gap between precision medicine and real-time adaptation (42). For instance, exposome-based AI models (43) could predict migraine onset based on air quality fluctuations (44) and dynamically adjust preventive pharmacotherapy (45) or neuromodulation. Similarly, circadian-aligned chronotherapy could optimize antidepressant administration times to enhance efficacy while minimizing migraine exacerbation (46, 47).

### 1.4 Challenges and feasibility considerations

While the Hybrid Digital-4E Strategy presents a promising direction, several challenges remain. AI-driven models (48) may inherit biases from training datasets, leading to inaccurate treatment recommendations for underrepresented populations. Addressing bias requires diverse, population-wide datasets and transparent, interpretable AI systems (49). The integration of real-time biometrics, digital phenotyping, and exposome tracking raises concerns about data security and patient autonomy (50). Federated learning and decentralized data models (51) could provide privacy-preserving AI frameworks. The transition from theoretical models to clinical implementation requires prospective validation studies. While AI-driven interventions show promise, regulatory pathways for approvals remain unclear. Digital health solutions must also align with patient behavior and clinical workflow to ensure long-term adherence (52). Human-AI collaboration (53) (e.g., AI-assisted therapy rather than full automation) may enhance engagement.

### 1.5 Bridging the gap: a research roadmap

By transitioning from categorical treatment approaches (54) to a dynamic, real-time adaptive framework, this paper presents a roadmap for the future of personalized migraine-depression management. The research roadmap explores key innovations, including integration for biomarker-driven precision treatment, neuromorphic AI for real-time, closed-loop therapy, adaptive chronotherapy for circadian-aligned interventions, and VR-based neuroplasticity training for symptom modulation. The coexistence of migraine and depression presents significant clinical challenges, as current treatment paradigms often fail to address their interconnected pathophysiology. While traditional pharmacological and behavioral therapies have provided symptomatic relief, they remain largely reactive and static. This paper aligns with the emerging focus on comorbidity-driven, precision-based interventions by proposing an AI-driven, real-time therapeutic framework. By integrating adaptive neuroadaptive therapy, multi-omics insights, and digital phenotyping, the Hybrid Digital-4E Strategy offers a next-generation approach that goes beyond classic treatments, providing a novel precision medicine strategy for managing migraine-depression comorbidity.

## 2 Theoretical foundation: the Hybrid Digital-4E Model

Traditional cognitive frameworks have long centered on the brain as the primary locus of thought, perception, and behavior. However, modern cognitive science has evolved beyond this reductionist view, recognizing that cognition is not confined to the brain alone but is instead shaped by interactions between the brain, body, environment, and external technological tools (55). This shift is encapsulated in the 4E model of cognition, which posits that cognition is Embodied, Embedded, Enactive, and Extended (56). This perspective offers a paradigm shift in understanding migraine-depression comorbidity, as it acknowledges the bidirectional interactions between neurophysiological states, behavioral patterns, environmental influences, and digital health interventions. By incorporating the principles of 4E cognition into a digital therapeutic framework, a real-time, adaptive, and personalized model can be developed, one capable of responding dynamically to the multifaceted nature of migraine and depression. In such model, the integration of AI-driven closed-loop therapy, exposome tracking, multi-omics analysis, and neuromorphic AI, leading to iterative validation and clinical implementation.

### 2.1 The 4E cognition framework and its role in migraine-depression treatment

The 4E cognition framework represents a fundamental shift from brain-centered symptom management to an integrative, interactive system where cognition, physiology, behavior, and environment continuously shape treatment outcomes (57). Each principle within the 4E model offers unique insights for precision medicine (58), particularly when integrated with AI-driven closed-loop therapy, digital phenotyping, and exposome tracking. Table 1 outlines each principle and its relevance to AI-driven closed-loop therapy, digital phenotyping, and exposome tracking, demonstrating how personalized, real-time interventions can surpass the limitations of traditional static treatment models.

**Table 1:** The 4E cognition framework and its relevance to migraine-depression management.

4E principle	Relevance to migraine-depression management
Embodied cognition	Bodily processes – such as the gut-brain axis, autonomic nervous system, and inflammatory responses – play a critical role in migraine and depression. AI-driven wearables can monitor heart rate variability (HRV), skin conductance, and inflammatory biomarkers, allowing for real-time, personalized interventions.
Embedded cognition	Cognition is influenced by external environmental and social factors, including air pollution, dietary habits, and circadian disruptions. Traditional treatment models often overlook these influences. AI-driven exposome tracking enables continuous assessment and adaptation of treatment strategies based on environmental inputs.
Enactive cognition	Cognition is an active and dynamic process, rather than a passive response. Digital therapeutics, such as VR-based neurofeedback and biofeedback, enable patients to actively engage with cognitive and sensory stimuli, helping to retrain maladaptive neural circuits associated with migraine and depression.
Extended cognition	Cognitive processes extend beyond the brain, incorporating external tools and digital health technologies. AI-driven closed-loop systems act as external cognitive regulators, continuously adjusting behavioral and pharmacological treatments based on real-time biometrics and environmental conditions.

By integrating 4E cognition principles into an AI-powered framework, personalized treatment models can adapt dynamically to individual variability, physiological states, and environmental influences, overcoming the static and reactive limitations of conventional treatment models.

### 2.2 Key components of the Hybrid Digital-4E Model

#### 2.2.1 AI-driven closed-loop therapy and digital phenotyping

Traditional trial-and-error treatment models rely on static diagnoses and generalized treatment regimens. In contrast, AI-driven closed-loop therapy enables continuous monitoring of real-time biometrics and dynamically adjusts interventions based on the patient’s digital phenotype – a high-resolution, continuously evolving representation of their health profile.

#### 2.2.2 Exposome integration for environmental and behavioral insights

The exposome is a critical yet underexplored determinant of migraine and depression. Chronic exposure to air pollutants, irregular sleep–wake cycles, noise pollution, and urban stressors can exacerbate symptoms and undermine treatment efficacy. The Hybrid Digital-4E Model leverages AI-powered wearable sensors, geospatial analytics, and behavioral tracking to implement proactive interventions before symptoms escalate.

#### 2.2.3 Multi-omics integration for personalized treatment

Genetics, proteomics, metabolomics, and microbiome variations influence individual treatment responses. The Hybrid Digital-4E Model integrates multi-omics data analysis to predict pharmacological responses to antidepressants or migraine medications, identify gut-brain interactions that may drive inflammation and neurovascular dysregulation, and enable targeted lifestyle and dietary interventions for precision medicine.

#### 2.2.4 Adaptive chronotherapy for circadian-aligned treatment

Circadian misalignment is a well-documented driver of migraine and depression, yet treatment approaches remain poorly aligned with individual biological rhythms. The Hybrid Digital-4E Model optimizes treatment timing to reduce medication side effects by aligning with natural hormonal cycles and improve mood regulation and sleep architecture.

#### 2.2.5 Neuromorphic AI for real-time adaptation

Neuromorphic AI mimics biological neural networks, allowing ultra-fast real-time decision-making. Unlike conventional AI, neuromorphic computing processes multi-modal health data instantaneously, optimizing personalized interventions with unprecedented efficiency.

#### 2.2.6 VR-based neuroplasticity training

VR-based cognitive and sensory therapy can retrain maladaptive pain and mood circuits through immersive neuroplasticity exercises. Unlike traditional CBT, VR therapies can dynamically adjust based on real-time neurophysiological feedback and enhance patient engagement through interactive, gamified therapy.

Collectively, the Hybrid Digital-4E Model represents a fundamental shift in migraine-depression therapy, integrating AI-driven adaptation, exposome-aware interventions, and multi-omics insights into a personalized, real-time treatment paradigm. Recognizing both its transformative potential and the challenges ahead, this framework offers a scientifically rigorous yet visionary roadmap for next-generation neurological and psychiatric care.

## 3 How the Hybrid Digital-4E Strategy works: a hypothetical implementation

The Hybrid Digital-4E integrates neuroadaptive therapy, multi-omics insights, and digital phenotyping into a real-time, AI-driven precision medicine approach for managing migraine-depression comorbidity. This closed-loop system continuously monitors, predicts, and adjusts treatment interventions based on multi-source data inputs, ensuring a highly personalized and dynamic approach.

### 3.1 Continuous monitoring: digital phenotyping and multi-omics insights

The system passively and actively collects real-time physiological, behavioral, and environmental data to detect early symptom patterns. Key components include:

- Digital Phenotyping: Wearable devices, smartphone interactions, speech/language analysis, social behavior tracking, and sleep/wake cycle monitoring.
- Multi-Omics Integration: Genomics, proteomics, metabolomics, and microbiome analysis provide insight into genetic predisposition, metabolic markers, and gut-brain interactions.
- Environmental and Exposome Tracking: The system monitors air pollution, circadian disruptions, diet, urban stressors, and temperature fluctuations to identify external migraine-depression triggers.

By integrating multi-layered data, the system recognizes individualized triggers such as inflammatory cytokine spikes, circadian misalignment, and behavioral shifts indicative of mood disturbances.

### 3.2 AI-driven neuroadaptive therapy (closed-loop adjustment)

Using neuromorphic AI and deep learning models, the system dynamically detects anomalies and predicts symptom onset, enabling real-time intervention. The closed-loop mechanism ensures treatments are continuously optimized. Key adaptive interventions include:

- Medication Optimization: Adjusts the timing and dosage of antidepressants or migraine treatments in alignment with circadian rhythms and metabolic profiles to minimize side effects and enhance efficacy.
- Neuromodulation and Biofeedback: If stress-induced migraine risk is detected, the system activates non-invasive neurostimulation (e.g., transcranial stimulation or vagus nerve modulation) to preemptively reduce symptoms.
- Cognitive and Behavioral Interventions: When depressive symptoms are identified through digital phenotyping (e.g., reduced communication, cognitive slowing), the system initiates AI-driven cognitive behavioral therapy (CBT), guided mindfulness, or VR-based neuroplasticity training.
- Exposome-Aware Therapy: Treatment recommendations dynamically adjust based on environmental triggers such as poor air quality or sleep disruption, mitigating symptom onset through personalized lifestyle modifications.

### 3.3 Real-time, personalized intervention strategy

Unlike static treatment models, the Hybrid Digital-4E Strategy continuously refines interventions based on real-time patient response. An example scenario is predicted migraine onset: AI detects an inflammatory biomarker spike combined with environmental stressors (e.g., high humidity, poor sleep) and advises preemptive neuromodulation and dietary adjustments to mitigate inflammation. It might also be related to an early detection of depression symptoms, where the system identifies reduced speech engagement and changes in digital interactions, prompting activation of VR-based mood therapy and light therapy adjustments to stabilize circadian rhythms. Another scenario can be related to detection of poor medication response, where pharmacogenomic data can reveal suboptimal metabolism of an antidepressant, prompting a recommendation for dose adjustment or medication switch, eliminating the trial-and-error process.

### 3.4 Scalability and integration into clinical workflows

The model is designed for seamless integration into clinical practice through, e.g., smartphone and wearables integration, where patients interact with a mobile interface that connects with wearables to deliver personalized interventions. Healthcare providers receive AI-generated insights and real-time treatment recommendations on a clinical dashboard, ensuring human oversight. AI-driven alerts notify clinicians of high-risk patients, facilitating timely intervention, enabling remote monitoring and telemedicine support.

**Figure 1:**
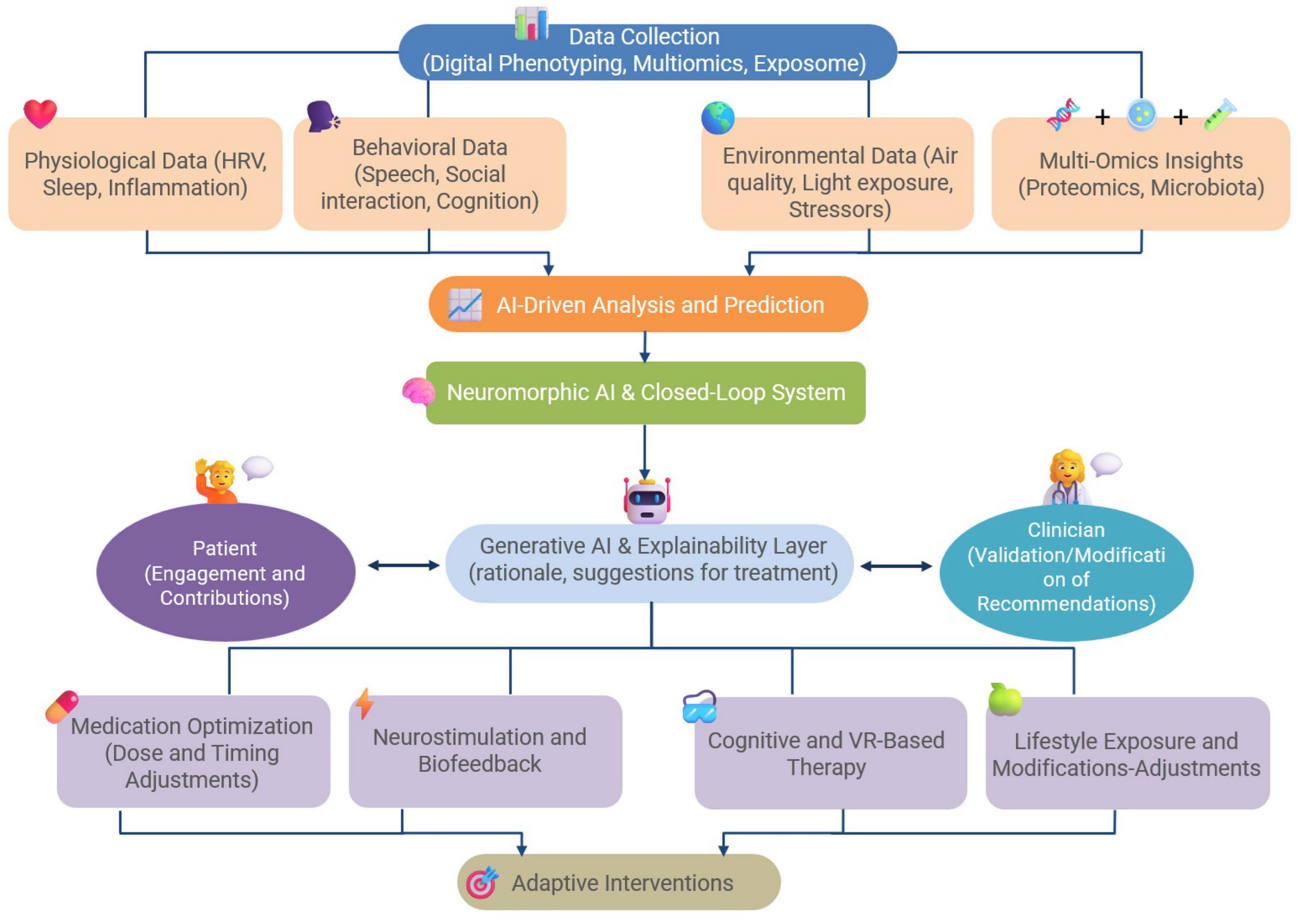
A conceptual diagram of the Hybrid Digital-4E Strategy for the management of comorbid migraine and depression.

The Hybrid Digital-4E Strategy revolutionizes migraine-depression care by shifting from reactive, symptom-based treatment to an adaptive, real-time intervention framework. It eliminates trial-and-error pharmacology by adapting interventions in real time based on patient variability while combines physiological, behavioral, environmental, and omics data to create an unprecedented level of treatment precision. It proactively intervenes before symptoms escalate, by reducing disease burden and improving patient outcomes. Therefore, the Hybrid Digital-4E Strategy represents a fundamental shift in migraine-depression management by bridging the gap between traditional pharmacology and next-generation digital therapeutics, this model offers a personalized, scalable, and highly adaptable treatment framework for comorbid neurological and psychiatric disorders. If successfully validated, this approach could set a new standard for individualized migraine-depression therapy, transforming both clinical and patient-driven care. Figure 1 depicts a conceptual diagram of the Hybrid Digital-4E Strategy for migraine-depression management.

## 4 Discussion

The Hybrid Digital-4E Model or strategy represents a transformative approach to migraine-depression treatment. Although this model presents substantial opportunities for improving patient outcomes, at least hypothetically as it is proposed here, several challenges, risks, and limitations must be critically examined to ensure clinical feasibility, ethical integrity, and regulatory compliance. This discussion evaluates the model’s strengths and innovations in comparison to existing treatment paradigms, key open research questions and future directions, clinical applications and testability of the model, and challenges and ethical considerations in real-world deployment.

### 4.1 Strengths and innovations of the model

The Hybrid Digital-4E Model presents several key innovations that distinguish it from traditional migraine and depression treatment approaches. Unlike conventional treatments that rely on static diagnoses and symptom-based interventions, this model offers a dynamic and adaptive approach that continuously adjusts therapeutic strategies based on real-time biometrics, behavioral patterns, and environmental data. By incorporating exposome tracking, the model surpasses traditional biopsychosocial approaches, allowing for personalized, contextualized interventions that account for external triggers. Moreover, the use of machine learning, neuromorphic AI, and multi-omics profiling enables high-precision medicine, optimizing both pharmacological and non-pharmacological interventions at an individualized level. Additionally, the model bridges neurology and psychiatry, recognizing migraine and depression as interconnected conditions rather than isolated disorders. This unified framework helps avoid fragmented care, providing a forward-looking, scientifically rigorous strategy for enhancing patient outcomes. Table 2 highlights the distinct features of the Hybrid Digital-4E Model compared to existing AI-driven digital health solutions for migraine and depression.

**Table 2:** Comparison of Hybrid Digital-4E Model with existing AI-based interventions.

Feature	Hybrid Digital-4E Model	Migraine tracking apps (e.g., migraine buddy, curelator)	Closed-Loop Neurostimulation (e.g., Nerivio, Cefaly)	Digital Phenotyping (e.g., Mindstrong, Koa Health)
Real-time, AI-driven closed-loop therapy	Supports real-time, adaptive interventions	Primarily retrospective symptom tracking	Limited to neurostimulation without broader adaptive therapy	Focuses on passive behavioral data collection, not real-time adjustments
Integration of exposome and environmental triggers	Includes exposome tracking for air quality, sleep patterns, and stressors	Some apps track weather or sleep, but lack comprehensive exposome analytics	No integration of environmental data	Does not consider environmental or lifestyle influences
Multi-omics data integration for precision treatment	Incorporates genetic, metabolic, and microbiome data to personalize treatment	No integration of multi-omics data	No biological data integration	No use of multi-omics analysis
Adaptive chronotherapy for circadian-aligned treatment	Uses AI to align treatments with individual circadian rhythms	No circadian-based treatment adjustments	No adaptation to biological rhythms	Can track sleep patterns but does not dynamically adjust treatment timing
Personalized AI-based cognitive training and VR therapy	Integrates VR-based neuroplasticity training and biofeedback for symptom modulation	No interactive cognitive training	No cognitive therapy integration	Some platforms offer CBT-based interventions but lack VR-based neuroplasticity training
Neuromorphic AI for real-time decision making	Uses neuromorphic AI to continuously optimize interventions	Does not use AI-driven decision-making	Not based on neuromorphic AI; limited to device-based stimulation	Does not incorporate AI-driven dynamic treatment adjustments
Scalability and remote monitoring integration	Designed for integration with mobile apps, wearables, and home-based monitoring	Available as mobile applications with some tracking features	Limited scalability beyond device use	Can integrate with wearables and mobile platforms

The Hybrid Digital-4E Model stands apart from existing solutions by integrating AI-driven, real-time adaptive interventions, multi-omics insights, exposome-aware analytics, and neuromorphic AI for real-time treatment modulation, capabilities that are currently absent in standalone migraine apps, neurostimulation devices, or digital phenotyping solutions.

### 4.2 Future research directions and open questions

While the Hybrid Digital-4E Model offers a groundbreaking shift in treatment, several key research questions remain. For instance, it is not yet known how neuromorphic AI can be validated for clinical decision-making. Unlike traditional machine learning models, neuromorphic AI processes data dynamically (59). Clinical validation studies will therefore be required to establish its reliability in real-world healthcare applications. Another open question is the identification of optimal biomarkers for digital phenotyping. Future research must determine the most predictive physiological and behavioral markers that correlate with migraine-depression interactions. Additionally, current regulatory frameworks are not designed for adaptive, closed-loop AI interventions, necessitating the creation of new validation pathways tailored for dynamic healthcare models. It is important to delineate a framework that enables regulatory agencies to evaluate real-time AI-driven treatments.

Longitudinal studies should also explore how continuous AI-based intervention affects neuroplasticity, medication adherence, and patient outcomes over extended periods. A crucial but unresolved question remains regarding the long-term effects of AI-driven adaptive therapy. Ensuring that AI-driven therapy remains accessible, equitable, and bias-free is both an ethical and practical challenge (60), requiring transparent AI development and diverse population datasets. Another key question that must be addressed is whether AI-driven treatment will reduce healthcare disparities or exacerbate them (61).

Historically, migraine and depression have been managed through separate pharmacological and behavioral strategies, with treatments such as antidepressants (SSRIs, SNRIs, and TCAs), CGRP antagonists, and neuromodulation techniques being employed in isolated frameworks. While these approaches have demonstrated efficacy, they do not account for the dynamic, bidirectional influences between migraine and depression. In contrast, emerging digital health innovations (62, 63), including wearable sensors, AI-driven therapy, and real-time closed-loop interventions, offer novel, integrative pathways for treatment. The Hybrid Digital-4E Model builds upon these advancements by creating a unified, adaptive framework that continuously adjusts therapeutic interventions based on real-time biometrics, exposome data, and neurocognitive insights. Future research should focus on validating this model within comorbidity-specific clinical trials, ensuring that novel AI-driven treatments are rigorously compared against existing standard-of-care therapies.

While this paper focuses specifically on migraine-depression comorbidity, the Hybrid Digital-4E Strategy is designed as a scalable and adaptable framework. The integration of real-time biometrics, exposome tracking, and adaptive AI could be equally relevant for other common migraine comorbidities, including sleep disorders, gut-brain axis dysfunctions, metabolic syndromes, menstrual-related symptoms, and broader psychiatric conditions such as anxiety or bipolar disorder. Future iterations of the model could be tailored to these comorbidity clusters, offering a modular approach to precision migraine care.

### 4.3 Potential clinical applications

The Hybrid Digital-4E Model is designed to be clinically actionable, enabling direct implementation into neurological and psychiatric care. Key applications include precision-based pharmacology, where AI-driven multi-omics integration can predict patient-specific responses to migraine and depression medications, reducing trial-and-error in treatment selection. Another implication is preemptive migraine and depression management, where AI-driven early warning systems could predict migraine attacks and depressive episodes before they manifest, enabling preventative interventions. The model can be deployed via smartphone applications, wearable biosensors, and remote monitoring systems, allowing real-time, patient-specific treatment adjustments. Moreover, AI-driven VR-based biofeedback and cognitive training could personalize therapy in real-time, making interventions more interactive, immersive, and engaging. These applications represent only a few examples of how the model could seamlessly integrate into real-world clinical practice, moving beyond theory into evidence-based, scalable treatment innovations. In addition to clinical integration, patient engagement and adherence are key to the real-world success of the Hybrid Digital-4E Model. Patient involvement is central to the Hybrid Digital-4E Strategy. Through wearable sensors and mobile interfaces, patients contribute both passive and active data streams, enabling dynamic phenotyping and adaptive therapy. Engagement is supported by real-time feedback, personalized goal-setting, and gamified cognitive training modules, particularly within VR-based neuroplasticity therapy. Patients retain control over data sharing via opt-in mechanisms, promoting autonomy and trust. Adherence is continuously monitored through behavioral signals (e.g., usage frequency, interaction patterns), while the system dynamically adapts to enhance engagement and therapeutic alignment.

### 4.4 Testability and iterative validation approach

To ensure that the Hybrid Digital-4E Model is not merely theoretical but clinically viable, a stepwise, iterative validation approach should be adopted. Rather than attempting immediate large-scale implementation, testing should progress through multiple stages. One potential stepwise development could begin with initial validation through individual case reports and small patient cohorts to assess AI-driven adaptations and feasibility. The next stage could involve pilot feasibility trials and small-scale, prospective trials evaluating data accuracy, patient adherence, and treatment outcomes. Large-scale observational studies could then be conducted to assess real-world efficacy, usability, and unintended consequences. Finally, multi-center RCTs comparing Hybrid Digital-4E-based interventions with traditional treatments could be run to determine long-term effectiveness, safety, and scalability. This agile, iterative approach ensures that risks are minimized and clinical confidence is built progressively before full-scale implementation. Table 3 presents the validation process, beginning with small-scale studies and culminating in regulatory approval and clinical deployment.

**Table 3:** Iterative validation approach for the Hybrid Digital-4E Model.

Phase	Description
Case studies and case groups	Initial validation through individual case reports and small patient cohorts to assess AI-driven adaptations and feasibility.
Pilot feasibility trials	Small-scale, prospective trials evaluating data accuracy, patient adherence, and treatment outcomes.
Clinical validation in observational studies	Large-scale observational studies assessing real-world efficacy, usability, and unintended consequences.
Randomized controlled trials (RCTs)	Multi-center RCTs comparing Hybrid Digital-4E-based interventions to traditional treatments to determine long-term effectiveness, safety, and scalability.

As outlined in Table 3, feasibility testing in future studies is crucial. Even though the Hybrid Digital-4E Model presents a theoretically robust and interdisciplinary framework, future research must focus on empirical validation to assess its clinical feasibility, effectiveness, and scalability. Feasibility testing should begin with small-scale pilot studies to evaluate the model’s technical viability, patient adherence, and AI-driven treatment adaptation accuracy. To establish the model’s real-world applicability, future studies should conduct case studies and prospective pilot trials to assess how AI-driven closed-loop therapy interacts with real-time biometrics and digital phenotyping. Additionally, it is crucial to investigate the predictive accuracy of exposome tracking in detecting migraine and depressive episode triggers and validate neuromorphic AI’s decision-making reliability compared to existing clinical assessment methods. It is also highly relevant to assess patient acceptability, engagement, and usability through qualitative and quantitative patient feedback.

While this model remains conceptual, several analogous systems in other medical domains support its feasibility. Closed-loop AI systems are in use for diabetes (e.g., adaptive insulin pumps), and mental health applications are increasingly leveraging digital phenotyping (e.g., Mindstrong). Neuromodulatory BCIs have shown efficacy in neurological disorders like epilepsy and stroke recovery. These examples indicate that the foundational components of the Hybrid Digital-4E Strategy are already under clinical investigation, providing a pathway toward future integration.

### 4.5 Challenges and ethical considerations

AI-driven closed-loop healthcare models introduce unique regulatory challenges, particularly regarding safety, transparency, and clinical validation (60, 64). Unlike traditional static treatment paradigms, closed-loop AI continuously adjusts therapy based on real-time physiological, behavioral, and environmental data, raising concerns regarding regulatory approval pathways. Current regulations focus on predefined, static interventions rather than continuously adapting AI-driven systems. The Hybrid Digital-4E Model must align with emerging AI-in-medicine frameworks such as the FDA’s Software as a Medical Device (SaMD) and Digital Health Pre-Certification Program. In addition, the use of neuromorphic AI for real-time decision-making raises concerns about algorithmic explainability and potential black-box decision-making in clinical settings. Future AI-driven healthcare applications must adopt Explainable AI frameworks to ensure clinician oversight and accountability (65). Since the model integrates biometric tracking, digital phenotyping, and exposome analytics, it requires compliance with GDPR (Europe), HIPAA (United States), and evolving AI ethics regulations. Privacy-preserving AI methodologies (e.g., federated learning, decentralized data processing) should be explored to balance patient autonomy and data security.

The Hybrid Digital-4E Model must therefore undergo prospective clinical validation through observational studies and multi-center RCTs to meet regulatory safety and efficacy standards. Collaboration with regulatory agencies, digital health researchers, and clinical practitioners will be essential for ensuring ethical deployment and real-world integration. Several ethical and practical challenges (Table 4) must be addressed before the Hybrid Digital-4E Model can be widely implemented in clinical settings.

**Table 4:** Ethical and implementation challenges in AI-driven migraine-depression therapy and proposed solutions.

Challenge	Potential solutions
AI transparency and clinician oversight	Adoption of Explainable AI and clinician-in-the-loop models to maintain human oversight.
Healthcare disparities and accessibility	Development of affordable wearables and digital health subsidies to ensure widespread access.
Patient autonomy and privacy concerns	Implementation of opt-in/opt-out patient control mechanisms with GDPR compliance.
Regulatory hurdles in AI-driven medicine	Establishment of new validation pathways tailored for closed-loop AI interventions.

To address key implementation challenges, such as AI bias, hallucination, and data privacy, the Hybrid Digital-4E Model incorporates multiple safeguards. First, explainable AI (XAI) methods are prioritized to ensure that output remains transparent and interpretable, allowing clinicians to review and override system recommendations. This “human-in-the-loop” framework supports a collaborative decision-making process where AI functions in an assistive role rather than operating autonomously, enhancing both safety and clinician trust. To protect patient data and align with GDPR and global digital health regulations, privacy-preserving strategies such as federated learning, differential privacy, and homomorphic encryption may be employed. These allow decentralized model training and secure data handling without compromising individual privacy. Collectively, these methods support ethical, transparent, and clinically robust integration of AI into migraine-depression care, aligning with regulatory initiatives such as the EU AI Act and the FDA’s SaMD framework.

While clinician-in-the-loop frameworks offer a safeguard against AI-driven errors, they also present practical challenges. One key issue is workflow integration—clinicians already face substantial time pressure, and introducing AI-assisted tools must not add complexity or cognitive load. Additionally, trust in AI-generated outputs is a known barrier, particularly when decisions lack transparency. To overcome this, explainable AI (XAI) models that offer clear rationale for each recommendation can help clinicians make informed choices. Training and digital literacy are also essential, as providers must understand how to interpret and interact with AI insights without overreliance. Furthermore, alert fatigue must be avoided through adaptive, customizable dashboards that prioritize clinically actionable insights. Addressing these factors will be key to the successful deployment of clinician-augmented digital health systems.

Addressing these challenges proactively will be essential for responsible AI integration into healthcare, ensuring ethical implementation, equitable access, and patient-centered design (66, 67).

## 5 Concluding remarks

The Hybrid Digital-4E Model offers a visionary yet scientifically rigorous framework for transforming migraine and depression treatment. By integrating AI-driven closed-loop therapy, exposome tracking, and the principles of embodied, embedded, enactive, and extended cognition, this model provides a real-time, adaptive, and highly personalized approach that goes beyond static, symptom-based paradigms. This theoretical hypothesis-driven paper has outlined a research roadmap, emphasizing the need for interdisciplinary collaboration across neurology, psychiatry, digital health, and AI ethics to refine, validate, and ethically implement such innovation. By embracing a digital and 4E-informed paradigm, the management of co-occurring migraine and depression can shift from reactive, trial-and-error strategies to proactive, dynamic, and precision-based care.

To translate this model into clinical practice, future efforts must prioritize stepwise clinical validation, ensuring compliance with evolving healthcare standards in digital health ethics and regulations. Additionally, scalability and accessibility must be addressed to mitigate potential disparities in digital health adoption. If validated and successfully tested, the Hybrid Digital-4E Model has the potential to redefine migraine and depression management, ushering in a new era of truly personalized neurological and psychiatric care when these conditions co-occur.

This paper contributes to the evolving landscape of migraine-depression therapy by shifting treatment paradigms from static, symptom-based approaches to AI-driven, real-time, precision-based care. While classic therapies remain the foundation of treatment, the Hybrid Digital-4E Model introduces a novel, integrative strategy that leverages AI, digital phenotyping, and multi-omics precision medicine to enhance patient outcomes. If validated, this approach could bridge the gap between conventional pharmacological models and next-generation, personalized digital therapeutics, marking a significant advancement in the management of comorbid neurological and psychiatric disorders.

## Data Availability

The original contributions presented in the study are included in the article/supplementary material, further inquiries can be directed to the corresponding author.
